# Research on nitrogen transformation pathways of a thermophilic heterotrophic nitrifying bacterial consortium GW7

**DOI:** 10.3389/fmicb.2025.1578865

**Published:** 2025-06-30

**Authors:** Yongqi Ma, Jiali Wang, Yindi Zhang, Wenping Guan, Wenrui Qi, Xisheng Tai, Dong Lin, Rong He, Likun Sun, Aiwen Zhang

**Affiliations:** ^1^College of Animal Science and Technology, Gansu Agricultural University, Lanzhou, China; ^2^College of Urban Environment, Lanzhou City University, Lanzhou, China; ^3^College of Pratacultural Gansu Agricultural University, Lanzhou, China; ^4^Yuzhong Modern Agricultural Investment Development Co., Ltd., Lanzhou, China; ^5^Gansu Provincial Animal Husbandry Technology Promotion Station, Lanzhou, China

**Keywords:** thermophilic heterotrophic nitrifying bacteria, bacterial consortium, response surface methodology, nitrogen transformation, nitrogen metabolic pathways

## Abstract

**Introduction:**

High-temperature heterotrophic nitrifying bacteria play a crucial role in the thermophilic phase of aerobic composting by effectively converting reduced ammonia sources. This process reduces ammonia emissions and contributes to nitrogen fixation, thus showcasing a high potential for application. The study aims to isolate and characterize a high-temperature-tolerant heterotrophic nitrifying bacterial consortium to enhance nitrogen transformation during aerobic composting.

**Methods:**

An excellent high-temperature-tolerant heterotrophic nitrifying bacterial consortium, designated GW7, was enriched from compost samples at elevated temperatures. The bacterial consortium was cultured under varying conditions to determine optimal cultivation parameters. Response surface methodology (RSM) experiments were conducted to find the best conditions for ammonia and nitrate nitrogen utilization. Enzymatic assays were carried out to measure the specific activities of key enzymes, including glutamine synthetase (GS), glutamate dehydrogenase (GDH), glutamate synthetase (GOGAT), ammonia monooxygenase (AMO), and hydroxylamine oxidoreductase (HAO).

**Results:**

At 55°C, the GW7 consortium demonstrated a utilization efficiency of 79.97% for ammonia nitrogen (NH₄+-N) (400 mg/L) and 21.18% for nitrate nitrogen (NO₃^−^-N) (400 mg/L). Response surface methodology identified the optimal cultivation conditions for GW7 as follows: sodium succinate as the carbon source, a C/N ratio of 15:1, a temperature of 53°C, initial pH of 6, and a rotation speed of 200 r/min. Under these conditions, the NH₄^+^-N utilization efficiency increased to 87.80%. Enzymatic assays showed that the specific activities of GS, GDH, and GOGAT were 0.392 U/mg, 0.926 U/mg, and 0.195 U/mg, respectively. Moreover, the specific activities of AMO and HAO were 1.459 U/mg and 0.701 U/mg, respectively.

**Discussion:**

The GW7 consortium demonstrated excellent nitrogen transformation capabilities, effectively utilizing ammonia nitrogen and contributing to the reduction of nitrogen losses in the aerobic composting process. The high enzymatic activities of key nitrogen-metabolizing enzymes, including AMO and HAO, support its role in heterotrophic nitrification. The proposed nitrogen conversion pathways, including ammonia assimilation, heterotrophic nitrification, and assimilatory nitrate reduction, highlight the versatile nitrogen metabolism of this bacterial consortium. The optimized cultivation conditions further enhance its practical application potential in mitigating nitrogen emissions during composting processes.

## Graphical abstract

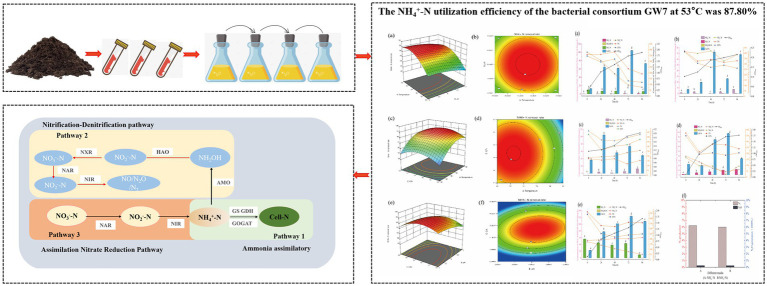

## Highlights

- A high-temperature-tolerant heterotrophic nitrifying bacterial consortium (GW7) was successfully obtained.
- Consortium GW7 demonstrated an exceptional NH₄^+^-N utilization efficiency of 87.80%.
- Consortium GW7 utilizes three distinct nitrogen transformation pathways: ammonia assimilation, heterotrophic nitrification, and assimilatory nitrate reduction.

## 1 Introduction

With the continuous advancement of socioeconomic conditions, the demand for livestock products has increased significantly, prompting the transition of livestock production toward intensive, large-scale systems. Consequently, the generation of livestock manure has markedly risen, leading to heightened risks of air, soil, and water pollution, and posing significant threats to environmental quality and public health (López-Sánchez et al., 2022). Aerobic composting has emerged as an effective and sustainable method for managing livestock manure (Zhao et al., 2023). Nevertheless, the elevated temperatures during aerobic composting intensify ammonia volatilization, resulting in substantial nitrogen losses typically ranging from 16 to 76%, with an average exceeding 40% (Li et al., 2018). Such nitrogen loss adversely affects the quality of the compost (Li et al., 2024). Heterotrophic nitrifying microorganisms, which utilize organic carbon sources under aerobic conditions, are capable of oxidizing reduced nitrogen forms such as ammonia/ammonium and organic nitrogen into hydroxylamine, nitrate, or nitrite. Integrating these microorganisms into composting processes has demonstrated enhanced nitrogen conversion efficiency (Wang et al., 2024a). Additionally, these microorganisms exhibit rapid propagation and robust adaptability to varying environmental conditions, making them ideal candidates for microbial inoculants aimed at improving nitrogen retention during composting (Ye et al., 2022; Xu et al., 2022). However, mesophilic nitrifying bacteria generally lose their activity or experience reduced ammonia oxidation capabilities during the thermophilic composting phase, compromising their effectiveness in sustaining nitrogen conversion and retention (Zhao et al., 2020). Therefore, the development and application of thermophilic nitrifying microorganisms are critical for mitigating nitrogen losses during the high-temperature stages of aerobic composting.

Previous studies have indicated that isolating thermotolerant nitrifying bacteria from aerobic compost and applying them during the composting process can effectively enhance nitrification and reduce nitrogen loss, presenting a promising strategy for improving compost quality (Kuroda et al., 2017; Zhao et al., 2020; Wang et al., 2024b). Researchers have therefore isolated and characterized several thermophilic heterotrophic nitrifying bacterial strains. For instance, *Brevibacillus agri* N2 demonstrated an ammonia nitrogen (NH₄^+^-N) utilization rate of 0.63 (mg/L h) at 60°C, with an initial NH₄^+^-N concentration of 99.64 mg/L (Zhao et al., 2022a). Similarly, *Gordonia paraffinivorans* N52 exhibited an NH_4_^+^-N utilization rate of 0.7 (mg/L h) under comparable temperature conditions (Zhao et al., 2022b). Compared with bacterial strains, microbial consortia enriched through repeated subculturing often exhibit superior efficiency, functional stability, and performance due to their microbial diversity and synergistic interactions, resulting in better practical outcomes (Han et al., 2024). Consequently, it is crucial to screen and construct functionally stable thermophilic nitrifying consortia. However, there remains a notable gap in the exploration and detailed understanding of the underlying mechanisms driving nitrogen transformation and retention by these consortia during high-temperature composting.

Furthermore, nitrogen loss during composting is closely linked to the intensity and interaction of various biological reactions, including ammonium oxidation and ammonium assimilation, predominantly mediated by thermophilic nitrifying microbial consortia. These reactions directly influence nitrogen transformation pathways by altering the rates and directions of nitrogen conversion processes, and affect the stability of nitrogen forms within compost by changing the balance between different nitrogen compounds (Maeda et al., 2011). Previous studies have highlighted the complexity and diversity of nitrogen transformation routes utilized by heterotrophic nitrifying bacteria, making this area a current focal point and challenging research topic. Known nitrogen transformation pathways primarily include: (1) heterotrophic nitrification-aerobic denitrification (HNAD), which follows the sequential route (1) NH₄^+^ → NH₂OH → NO₂^−^ → NO₃^−^ → NO₂^−^ → NO → N₂O → N₂; and (2) direct ammonia oxidation (Dirammox), which proceeds via NH₄^+^ → NH₂OH → NO→N₂O → N₂ (Song et al., 2021). In addition, certain heterotrophic nitrifiers possess assimilatory nitrate reduction to ammonium (ANRA) and dissimilatory nitrate reduction to ammonium (DNRA) pathways, enabling them to reduce NO₃^−^ to NO₂^−^, subsequently generating NH₄^+^ that is assimilated for bacterial growth (Zhu et al., 2023a). Under conditions of elevated nitrate concentration, many heterotrophic nitrifiers utilize aerobic denitrification pathways to alleviate metabolic stress, thereby facilitating sustained energy acquisition and microbial proliferation (Silva et al., 2019). However, elevated composting temperatures significantly impact the metabolic processes of heterotrophic nitrifiers by disrupting cellular structures, altering key enzymatic activities, and consequently, influencing nitrogen transformation pathways (Marietou, 2021). Despite these insights, the precise metabolic pathways and enzymatic mechanisms governing nitrogen transformations in thermophilic heterotrophic nitrifying bacteria remain inadequately characterized, and investigations of key enzyme activities involved in heterotrophic nitrification under high-temperature conditions are relatively limited. This knowledge gap restricts the practical application of thermophilic heterotrophic nitrifying consortia in composting processes.

Research on the nitrogen transformation pathways of thermophilic heterotrophic nitrifying bacterial consortia is currently limited. Most existing studies have focused on the nitrogen transformation capabilities of individual strains, while the nitrogen transformation mechanisms in complex microbial consortia under high-temperature conditions remain poorly understood. This study systematically investigates the nitrogen transformation pathways of the high-temperature heterotrophic nitrifying bacterial consortium GW7, thereby addressing this knowledge gap. Therefore, this experiment focuses on the thermophilic heterotrophic nitrifying consortium GW7, previously enriched and selected through repeated subcultures by our research team. The objectives of this research are: (1) to optimize cultivation conditions for this consortium, aiming to substantially enhance nitrification efficiency and achieve high-density microbial cultivation (≥10^8^ CFU/mL); (2) to analyze the consortium’s utilization efficiency and metabolic pathways for various inorganic nitrogen sources under optimized conditions; and (3) to elucidate the nitrogen transformation mechanisms of consortium GW7 by analyzing the activities of key enzyme activities involved in nitrogen metabolism. Collectively, this research aims to provide robust microbial resources and scientific evidence to support the practical application of thermophilic heterotrophic nitrifying consortia in reducing nitrogen losses during aerobic composting.

## 2 Materials and methods

### 2.1 The enrichment of bacterial consortium

In previous laboratory experiments, cattle and sheep manure compost samples collected during the thermophilic phase (above 55°C) served as isolation sources. Following enrichment and acclimation in a heterotrophic nitrification medium, repeated subculturing procedures yielded a stable microbial consortium (GW7) exhibiting highly efficient heterotrophic nitrification capabilities.

The initial NH₄^+^-N concentration was established at 100 mg/L at 55°C and subsequently increased in increments of 100 mg/L. Results indicated that consortium GW7 achieved optimal NH₄^+^-N utilization efficiency at a concentration of 400 mg/L. Further elevation of NH₄^+^-N concentrations beyond this threshold led to significant reductions in both microbial growth performance and nitrification efficiency.

The high-throughput sequencing analysis of the 16S rRNA gene V3–V4 regions revealed that the consortium was predominantly composed of *Aeribacillus* (82.6%). The V3–V4 hypervariable regions were amplified using specific primers (F: ACTCCTACGGGAGGCAGCA; R: GGACTACHVGGGTWTCTAAT). Paired-end sequencing was conducted on the Illumina NovaSeq platform by Biomarker Technologies (Beijing, China). The consortium was currently maintained at the College of Animal Science and Technology, Gansu Agricultural University, with storage conditions at −80°C. Detailed procedures for strain enrichment and isolation are provided in the Supplementary material.

### 2.2 Culture media

The bacterial consortium GW7 was enriched in LB medium, and its nitrogen conversion capacity was evaluated under different inorganic nitrogen sources using five distinct heterotrophic nitrification media, as previously described by Zhao et al. (2022b).

The Medium A (base medium) contained (g/L): (NH₄)₂SO₄ 1.89, sodium succinate (C₄H₄Na₂O₄) 20.18, and 50 mL of Vishniac’s salt solution, with an adjusted initial pH of 7.0.

Different nitrogen sources were substituted into the base medium to prepare the following experimental treatments:

Medium B (denitrification medium): KNO₃ (2.18 g/L); Medium C: KNO₃ (1.44 g/L) + NaNO₂ (0.98 g/L).
Medium D (heterotrophic nitrification and aerobic denitrification medium): (NH₄)₂SO₄ (0.94 g/L) + KNO₃ (1.44 g/L); Medium E (heterotrophic nitrification and aerobic denitrification medium): (NH₄)₂SO₄ (0.94 g/L) + NaNO₂ (0.98 g/L).
Vishniac’s salt solution contained (g/L): K₂HPO₄ 5.00, MgSO₄·7H₂O 2.50, NaCl 2.50, FeSO₄·7H₂O 0.05, and MnSO₄ 0.05.

### 2.3 Single factor experiments on the nitrification performance of the high-temperature-tolerant bacterial consortium

Single-factor experiments were conducted to investigate the effects of various carbon sources (glucose, sodium citrate, sodium succinate, sucrose, sodium acetate), C/N (5, 10, 15, 20, 25), pH levels (5, 6, 7, 8, 9, 10), temperatures (45°C, 50°C, 55°C, 60°C, 65°C), and shaking speeds (80 rpm, 120 rpm, 160 rpm, 180 rpm, 200 rpm) on the heterotrophic nitrification and nitrogen utilization performance of the high-temperature heterotrophic nitrifying consortium. Samples were collected every 12 h to measure optical density at OD₆₀₀. Following this, the supernatant was obtained through centrifugation to determine the concentrations of NH₄^+^-N, NO₂^−^-N, NO₃^−^-N. The NH₄^+^-N utilization efficiency was subsequently calculated. OD₆₀₀ was measured using spectrophotometry, NO₃^−^-N was quantified by Ultraviolet Spectrophotometry, NO₂^−^-N was measured using N-(1-Naphthyl) Ethylenediamine Spectrophotometry, and NH₄^+^-N was assessed using Sodium Nitroprusside Spectrophotometry (APHA, 2005).

### 2.4 Optimization of culture conditions using response surface analysis

The Design-Expert V8 software was utilized to create a 3-factor, 3-level Box–Behnken Design (BBD) experiment. The three primary factors influencing the NH₄^+^-N utilization efficiency by the consortium were identified. The response variable A was temperature, ranging from 50 to 60°C; B was initial pH, ranging from 5 to 7; and C was the C/N, ranging from 10 to 20. The NH₄^+^-N utilization efficiency was designated as the response variable. Regression analysis, significance testing, and analysis of variance (ANOVA) were employed to predict the optimal nitrogen utilization conditions for consortium. The factors and levels for the response surface design are presented in Table 1. Response surface methodology (RSM) was applied to optimize the conditions for NH₄^+^-N utilization by the consortium.

**Table 1:** Box–Behnken experimental design with three independent variables.

Test number	Level			NH₄^+^-N utilization efficiency
	Temperature/°C	pH	C/N	
1	55	7	10	76.55%
2	60	7	15	49.17%
3	55	6	15	87.13%
4	60	5	15	47.49%
5	60	6	20	55.88%
6	55	5	20	77.83%
7	50	7	15	77.22%
8	55	7	20	74.53%
9	50	6	20	77.09%
10	50	6	10	75.26%
11	55	5	10	75.39%
12	55	6	15	87.54%
13	55	6	15	86.98%
14	55	6	15	85.06%
15	50	5	15	76.53%
16	55	6	15	83.21%
17	60	6	10	51.83%

### 2.5 Study on the nitrification performance of the high-temperature-tolerant heterotrophic nitrifying bacteria consortium

The bacterial consortium was inoculated into different media at 1% inoculation volume under the following conditions: A: (NH₄)₂SO₄ as the sole nitrogen source; B: NaNO₂ as the sole nitrogen source; C: NaNO₂ + KNO₃ as nitrogen sources; D: (NH₄)₂SO₄ + KNO₃ as nitrogen sources; E: (NH₄)₂SO₄ + NaNO₂ as nitrogen sources. The cultures were incubated at 55°C and agitated at 180 rpm for 96 h. Samples were collected every 24 h, with uninoculated media serving as controls. All experiments were conducted in triplicate. The concentrations of optical density at OD₆₀₀, NH₄^+^-N, NO₂^−^-N, NO₃^−^-N, NH₂OH-N, TN, Cell-N, TDN were measured. The detection methods for OD₆₀₀, NO₃^−^-N, NH₄^+^-N, NO₂^−^-N were consistent with those described in section 1.3. NH₂OH-N was measured using Indirect Spectrophotometry, while TN was determined by Alkaline Potassium Persulfate Ultraviolet Spectrophotometry (Studt et al., 2020).

### 2.6 Detection of gaseous products

The bacterial consortium GW7 was inoculated into serum bottles containing media A and B at 1% inoculum. The bottles were aerated with pure oxygen for 10 min to eliminate N₂. After aeration, the three-way valve was closed to ensure that the gas inside the bottle remained isolated from the external environment. Cultures were incubated at 55°C and shaken at 180 rpm. Gas samples (100 mL) were collected at regular intervals over a 48-h period using a gas-tight syringe. Gas chromatography was used to determine the concentrations of N₂, N₂O, and O_2_ (Xie et al., 2023). N_2_O was measured using an Agilent 7890A gas chromatograph (Agilent Technologies Inc. of the United States), while N₂ and O₂ were measured by a GC-14C gas chromatograph (Shimadzu Corporation of Japan).

### 2.7 Enzyme activity assays

The bacterial consortium GW7 was inoculated into a heterotrophic nitrification medium and incubated at 53°C for 108 h. The bacterial suspension was centrifuged at 10,000 rpm and 4°C for 20 min, and the pellet was washed three times with 0.01 mol/L PBS (pH 7.4). After washing, the cells were resuspended, and the suspension was sonicated using an ultrasonic cell crusher (Scientz-IID, Ningbo Xinzhi Biotechnology Co., Ltd.) to obtain a cell-free extract. The extract was centrifuged again at 10,000 rpm and 4°C for 20 min, and the supernatant was collected as the crude enzyme solution. Enzyme activity assays for AMO, HAO, NIR, NAR, GS, GDH and GOGAT were conducted. The extract was centrifuged again at 10,000 rpm and 4°C for 20 min, and the supernatant was collected as the crude enzyme solution. Enzyme activity assays for AMO, HAO, NIR, NAR, GS, GDH and GOGAT were conducted using bacterial ELISA kits (Jiangsu Jingmei Biotechnology Co., Ltd.). The enzyme activities were determined based on the depletion of nitrogenous substrates, and each treatment was performed in triplicate. Enzyme activity was expressed as units per milligram of protein (U/mg protein). Protein concentration was measured using the Coomassie brilliant blue method. In the experimental design, this experiment included a control group and blank calibration to verify the accuracy and reliability of the results. The control group consisted of reaction systems without substrate, used to assess background enzyme activity; the blank calibration included reaction systems without enzyme, used to calibrate the zero point of the assay kit. All enzyme activity measurements were performed in triplicate to ensure the reliability of the data.

### 2.8 Analytical methods and calculation

The utilization efficiency and utilization rate of nitrogen compounds under different conditions were calculated as follows, methods referenced from Xia et al. (2020):


$$N\;\text{utilization efficiency}\;(\%)=(C_{0}h-C_{t}h)/C_{0}h\times 100\%$$

$$N\;\text{utilization rate}\;(\mathrm{mg}/L\;h)=(C_{0}h-C_{t}h)/t$$

where C_0_h, C_t_h are the initial and final concentrations of each nitrogen compound (e.g., NH₄^+^-N, NO₃^−^-N, NO₂^−^-N, NH₂OH-N) at 0 h and t h, respectively.


$$\mathrm{Gas}-N\;(\mathrm{mg}/L)={\mathrm{TN}}_{0}h-{\mathrm{TN}}_{t}h$$

$$\text{Cell}-N\;(\mathrm{mg}/L)=\text{Cell}-N_{t}h-\text{Cell}-N_{0}h$$

TN_0_h, TN_t_h: total nitrogen of 0 h, t h; Cell-N_t_h, Cell-N_0_h: cell nitrogen of 0 h, t h.

Data were subjected to one-way analysis of variance (ANOVA) using IBM SPSS Statistics 25 (SPSS Inc., United States). Duncan’s multiple range test was applied for *post hoc* comparisons when ANOVA revealed significant differences (*p* < 0.05). Results are expressed as mean ± standard deviation (SD). Statistical significance was defined at *p* < 0.05, whereas *p* > 0.05 was considered non-significant. Graphical representations were prepared using Origin 2018 (OriginLab, United States) based on mean and SD values.

## 3 Results

### 3.1 Effects of different environmental factors on the nitrification performance of the high-temperature-tolerant heterotrophic nitrifying consortium

#### 3.1.1 Effect of carbon source

**Figure 1:**
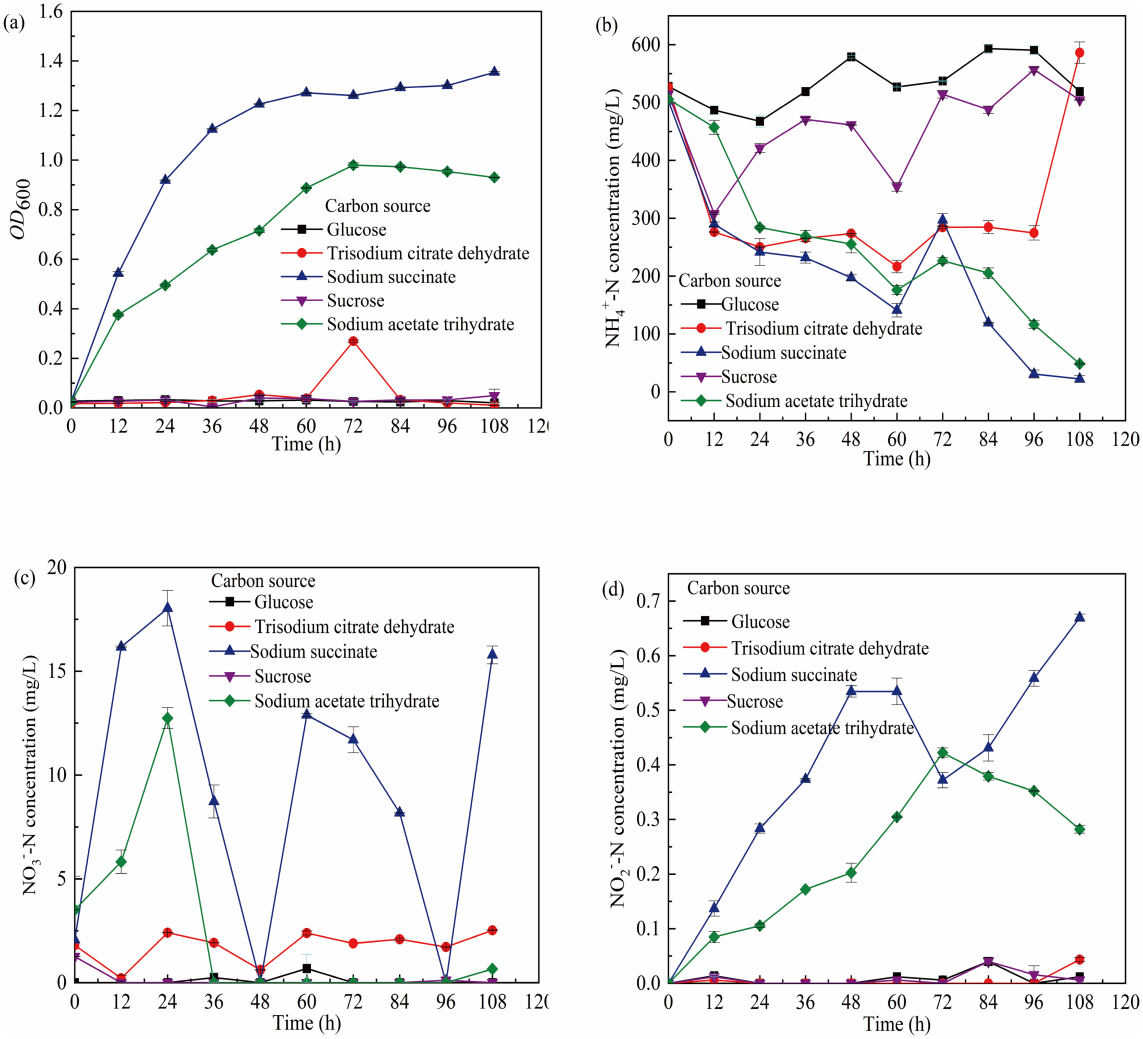
Growth of GW7 at 0–108 h under the influence of different carbon sources. **(a)** OD_600_ of mycelium. **(b)** Changes in NH₄^+^-N concentration. **(c)** Changes in NO₂^−^-N concentration. **(d)** Changes in NO₃^−^-N concentration.

Carbon sources, as essential energy and nutrient substrates for microbial growth, significantly influence the metabolic activity of heterotrophic nitrifying bacterial consortia (Zhang et al., 2022a). This study revealed differences in consortium growth and NH₄^+^-N removal efficiency across different carbon sources, under glucose, sucrose, and sodium citrate conditions, consortium growth was limited (OD₆₀₀ ≈ 0) with NH₄^+^-N removal efficiencies of only 0–2.00% (Figure 1a). Notably, the sodium citrate group exhibited NH₄^+^-N accumulation, likely due to nitrogen release from cell lysis. In contrast, when sodium succinate and sodium acetate served as carbon sources, consortium growth was significantly enhanced (OD₆₀₀ = 1.0), with NH₄^+^-N removal rates reaching 4.45 and 4.23 mg/(L h) respectively, and removal efficiencies as high as 95.59 and 90.41% (Figure 1b). Experimental data demonstrated the following carbon source performance ranking: sodium succinate > sodium acetate > sucrose > glucose > sodium citrate. The superior performance of sodium succinate may be attributed to its molecular structure being more readily metabolized by the bacterial consortium, effectively promoting energy acquisition, enhancing enzymatic activity, and optimizing nitrogen transformation pathways (Görke and Stülke, 2008). When sodium succinate was used as the carbon source, the concentration of NO₂^−^-N increased from 0 mg/L at 0 h to 0.663 mg/L at 108 h (Figure 1c); the concentration of NO₃^−^-N rised from 2.07 mg/L at 0 hours to 18.03 mg/L at 24 h, then begins to decrease, reaching 0 mg/L at 48 h, and then started to increase again, finally reached 15.79 mg/L (Figure 1d). This indicated that small molecular carbon sources are more favorable for GW7 to perform heterotrophic nitrification.

#### 3.1.2 Effect of pH

**Figure 2:**
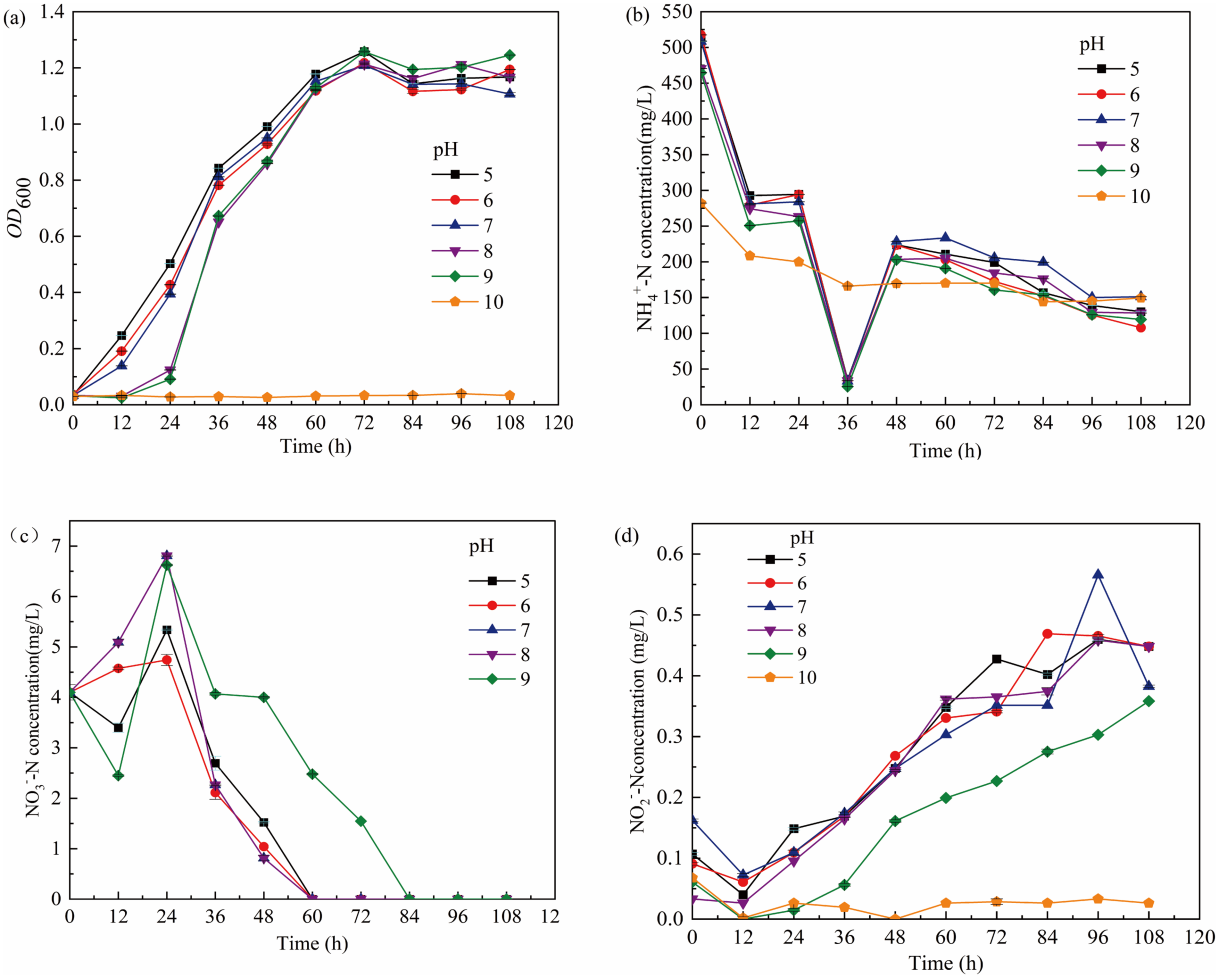
Growth of GW7 at 0–108 h under the influence of different pH. **(a)** OD_600_ of mycelium. **(b)** Changes in NH₄^+^-N concentration. **(c)** Changes in NO₂^−^-N concentration. **(d)** Changes in NO₃^−^-N concentration.

Most heterotrophic nitrifying bacteria thrive at a pH range of 7.0–7.5, with significant deviations impairing cell membrane integrity and metabolic activity (Zeng et al., 2020). The growth and metabolism of the heterotrophic nitrifying consortium were studied across a pH range of 5 to 9. At pH 5, 6, or 7, the consortium went through the following phases: lag (0–12 h), logarithmic growth (12–48 h), stationary (60–72 h), and decline phase (72–108 h). At pH 8 and 9, the lag phase was extended to 24 h, and the logarithmic growth phase was delayed by 12 h (Figure 2a). At 36 h, NH₄^+^-N utilization efficiencies at various pH levels were 74.40, 79.17, 70.30, 72.71, 74.31, and 47.02%, respectively. At pH 5, 6, or 7, the NH₄^+^-N utilization rates were 3.50 mg/(L h), 3.79 mg/(L h), and 3.19 mg/(L h), respectively (Figure 2b). The decrease in NO₃^−^-N concentration indicates conversion to NO₂^−^-N by nitrate reductase (Figure 2d), while the continued increase in NO₂^−^-N suggests ineffective utilization of NO₂^−^-N by the consortium (Figure 2c). These results show that the optimal pH range for the consortium’s growth and metabolism is between 5 and 7.

#### 3.1.3 Effects of carbon-to-nitrogen ratio

**Figure 3:**
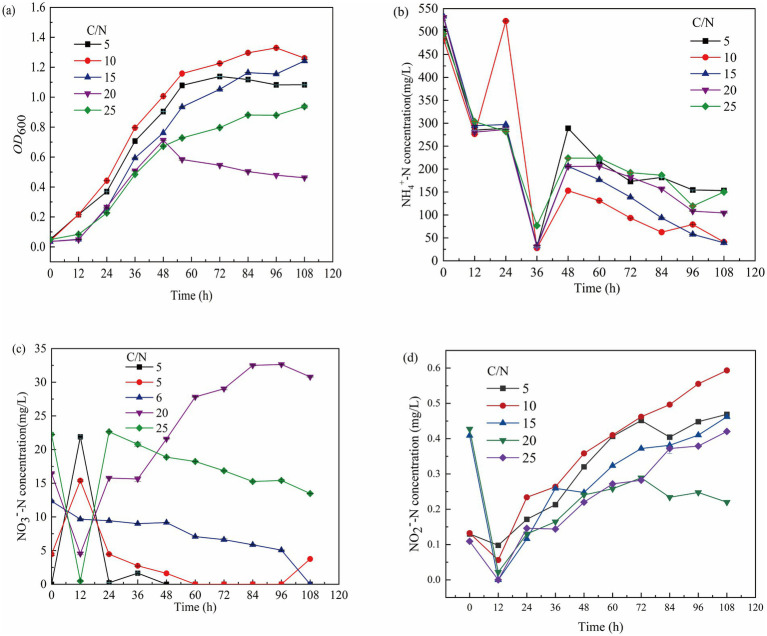
Growth of GW7 at 0–108 h under the influence of different C/N. **(a)** OD_600_ of mycelium. **(b)** Changes in NH₄^+^-N concentration. **(c)** Changes in NO₂^−^-N concentration. **(d)** Changes in NO₃^−^-N concentration.

The C/N ratio is a key factor in microbial nitrogen utilization, affecting the balance between electron donors and acceptors (Rajta et al., 2019). A C/N below 5 limits bacterial growth, while higher ratios promote selective use of carbon and nitrogen, influencing metabolic byproducts (Deng et al., 2020). Nitrifying bacteria have a wide adaptability to the carbon-to-nitrogen ratio (C/N), with an optimal range for growth between 5 and 20. An appropriate C/N can significantly accelerate the nitrification rate during the nitrification process. A C/N that is too low provides insufficient energy for the growth of nitrifying bacteria, while a C/N that is too high inhibits bacterial reproduction. As shown in Figure 3, during the first 12 h, bacteria rapidly utilized nutrients for growth at C/N ratios of 10 and 15, while growth was slower at C/N 5, 20, and 25 (Figure 3a). After 108 h, NH₄^+^-N utilization efficiencies were 91.52, 92.62, and 80.36% at C/N 10, 15, and 20, respectively (Figure 3b). At C/N 15, NO₃^−^-N concentration decreased from 12.34 mg/L to 0 mg/L, while at C/N 20 and 25, NO₃^−^-N accumulated to 14.35 mg/L and 8.77 mg/L, respectively (Figure 3d). These findings, consistent with Wang et al. (2023). Thus, the optimal C/N for the consortium’s growth and metabolism is between 10 and 20.

#### 3.1.4 Effects of temperature

**Figure 4:**
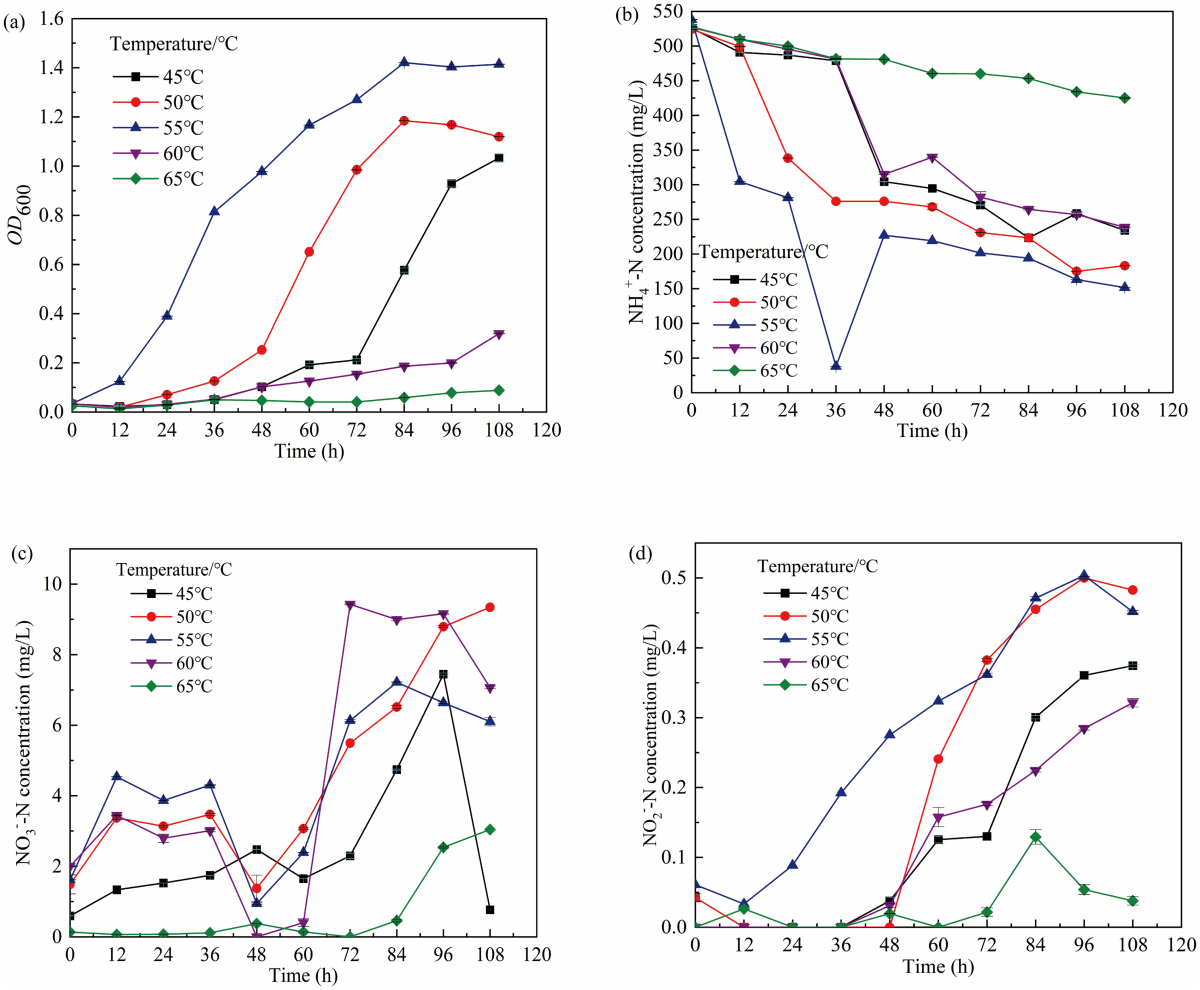
Growth of GW7 at 0–108 h under the influence of different temperature. **(a)** OD_600_ of mycelium. **(b)** Changes in NH₄^+^-N concentration. **(c)** Changes in NO₂^−^-N concentration. **(d)** Changes in NO₃^−^-N concentration.

Generally, high temperatures inhibit the growth and metabolism of nitrifying bacteria (Ganigu’e et al., 2007). The effects of temperature on the growth and metabolism of the nitrifying consortium are shown in Figure 4. At 45°C, 50°C, and 55°C, the consortium entered the logarithmic growth phase at 72 h, 48 h, and 12 h, respectively, indicating that lower temperatures delay growth. At 108 h, when the temperature was 60°C or 65°C, the OD₆₀₀ was below 0.2, and NH₄^+^-N utilization efficiencies at 50°C and 55°C were 65.06 and 71.83%, respectively. Even at 60°C, the utilization efficiency remained at 54.7%. At all temperatures (45°C, 50°C, 55°C, and 60°C), NO₂^−^-N accumulation exceeded 0.2 mg/L at 108 h. The highest NH₄^+^-N utilization efficiency of 71.83% was observed at 55°C, suggesting that the optimal temperature for the growth and metabolism of the consortium is between 50°C and 60°C.

#### 3.1.5 Effects of rotational speed

**Figure 5:**
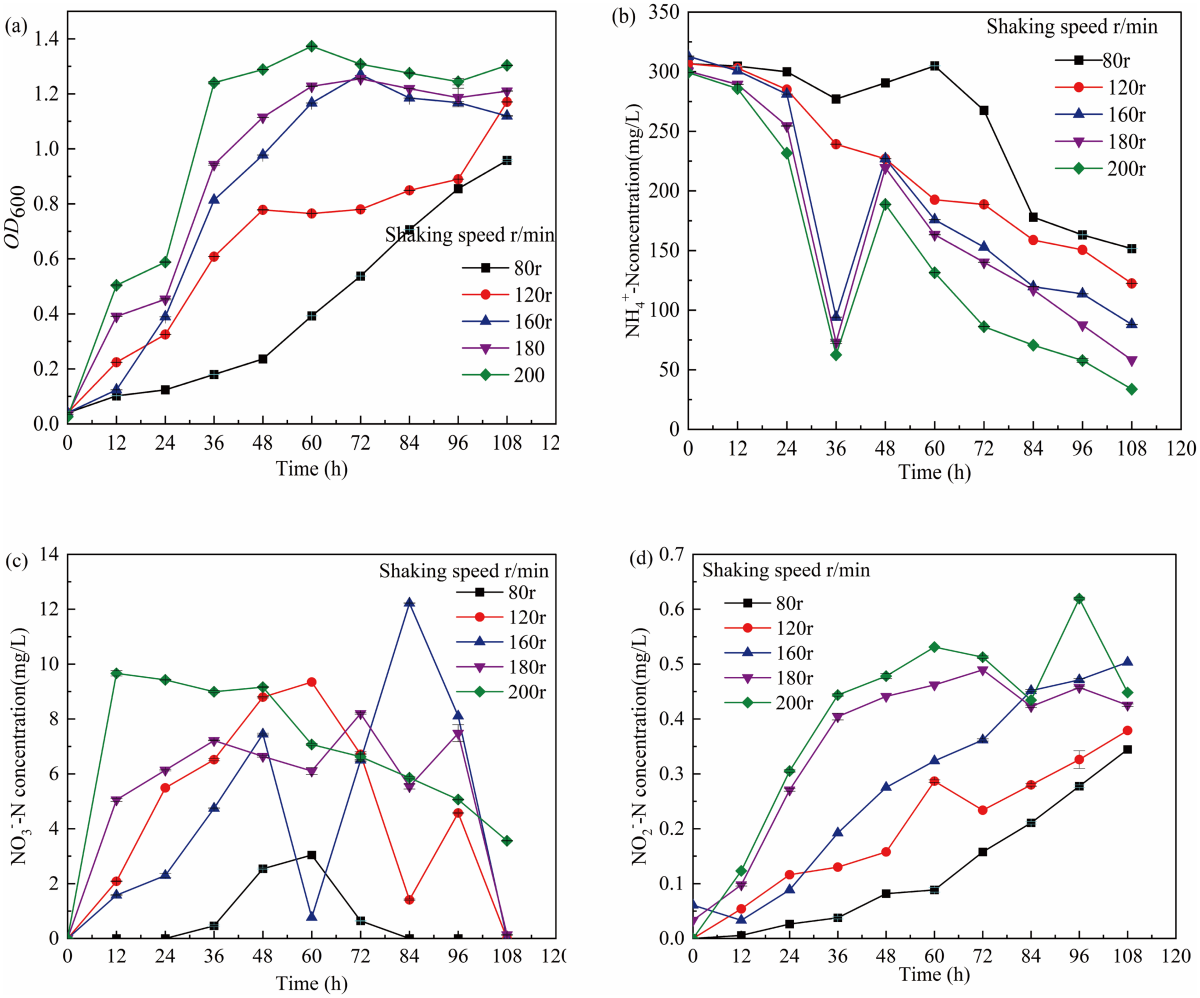
Growth of GW7 at 0–108 h under the influence of different rotating speeds was studied. **(a)** OD_600_ of mycelium. **(b)** Changes in NH₄^+^-N concentration. **(c)** Changes in NO₂^−^-N concentration. **(d)** Changes in NO₃^−^-N concentration.

The impact of shaking speed on the nitrogen utilization performance of nitrifying bacteria is primarily associated with oxygen transfer and mixing efficiency. An optimal shaking speed can enhance oxygen solubility and promote bacterial metabolic activities; however, excessively high shaking speeds may result in bacterial sedimentation and agitation, which can inhibit growth (Tong and Chen, 2009). Except for the 80 r/min condition, the OD₆₀₀ exceeded 1 at all other shaking speeds. Higher shaking speeds led to increased dissolved oxygen concentrations, accelerating bacterial growth (Figure 5a). After 108 h, NH₄^+^-N utilization efficiencies were 71.88, 80.62, and 88.73%, respectively (Figure 5b). A significant negative correlation was observed between NO₂^−^-N and NH₄^+^-N concentrations, with a decrease in NH₄^+^-N corresponding to an increase in NO₂^−^-N levels (Figures 5b,d). The maximum shaking speed used was 200 r/min, at which the NH₄^+^-N utilization efficiency reached 88.73%. As NH₄^+^-N decreased, the NO₃^−^-N content increased, which indicated that the process of nitrification was occurring(Figure 5c).

### 3.2 Optimization of the growth conditions for consortium using response surface methodology

#### 3.2.1 Regression model fitting and analysis of variance

Based on the results of the single-factor experiments, a quadratic model using Box–Behnken design was applied to further optimize the three parameters: C/N, initial pH, and temperature. The response values obtained from the experiments are shown in Table 1. To establish the relationship between the response and the independent variables, a quadratic model was constructed according to the recommendations of the Box–Behnken design. The equation is as follows:


$$\begin{array}{l} Y(\%)=+85.98-12.72A+0.02\mathrm{9B}+0.79C+0.25\mathrm{AB}+\\ \;0.55\;\mathrm{AC}-1.12\;\mathrm{BC}-17.22A^{2}-6.16B^{2}-3.75C^{2}. \end{array}$$

The determination coefficient *R*^2^ was 0.9866.

According to the principles of the Box–Behnken central composite design in response surface methodology, the NH₄^+^-N utilization efficiency at 108 h was selected as the response variable. The three key factors—temperature, C/N, and pH—were optimized by designing a three-factor, three-level experimental combination (Table 2). The adequacy and validity of the model were assessed through analysis of variance (ANOVA) (Hu et al., 2022). The ANOVA results for the regression model are presented in Table 2. As shown in Table 2, the model’s *p*-value was less than 0.0001, indicating that the model was highly significant and reliable. The quadratic terms A^2^ and B^2^ had *p*-values less than 0.01, indicating a highly significant effect on the response value, while the quadratic term C^2^ had a *p*-value less than 0.05, indicating a significant effect on the response value. The *F*-value for the regression model was 57.37, with a *p*-value less than 0.01, indicating an extremely significant level. The lack-of-fit term had an *F*-value of 2.57 (*p* > 0.05), indicating that the lack-of-fit was not significant. This indicated that the experimental error was small and that the regression model had a good fit to the experimental data.

**Table 2:** ANOVA test for response surface quadratic model (Y).

Source	Sum of squares	df	Mean square	*F*-value	*p*-value	Significant
Model	2872.26	9	319.14	57.37	<0.0001	**
A-Temperature	1293.73	1	1293.73	232.55	<0.0001	**
B-pH	0.01	1	0.01	0.00	0.9737	—
C-C/N	4.95	1	4.95	0.89	0.3772	—
AB	0.25	1	0.25	0.05	0.8379	—
AC	1.23	1	1.23	0.22	0.6524	—
BC	4.99	1	4.99	0.90	0.375	—
A^2^	1248.44	1	1248.44	224.41	<0.0001	**
B^2^	159.76	1	159.76	28.72	0.0011	**
C^2^	59.13	1	59.13	10.63	0.01386	*
Residual	38.94	7	5.56	—	—	—
Lack of fit	25.64	3	8.55	2.57	0.1921	—
Pure error	13.31	4	3.33	—	—	—
Corr. total	2911.20	16	—	—	—	—

*Indicates *p* < 0.05. **Indicates *p* < 0.01.

#### 3.2.2 Response surface analysis and determination of optimal nitrogen utilization conditions

**Figure 6:**
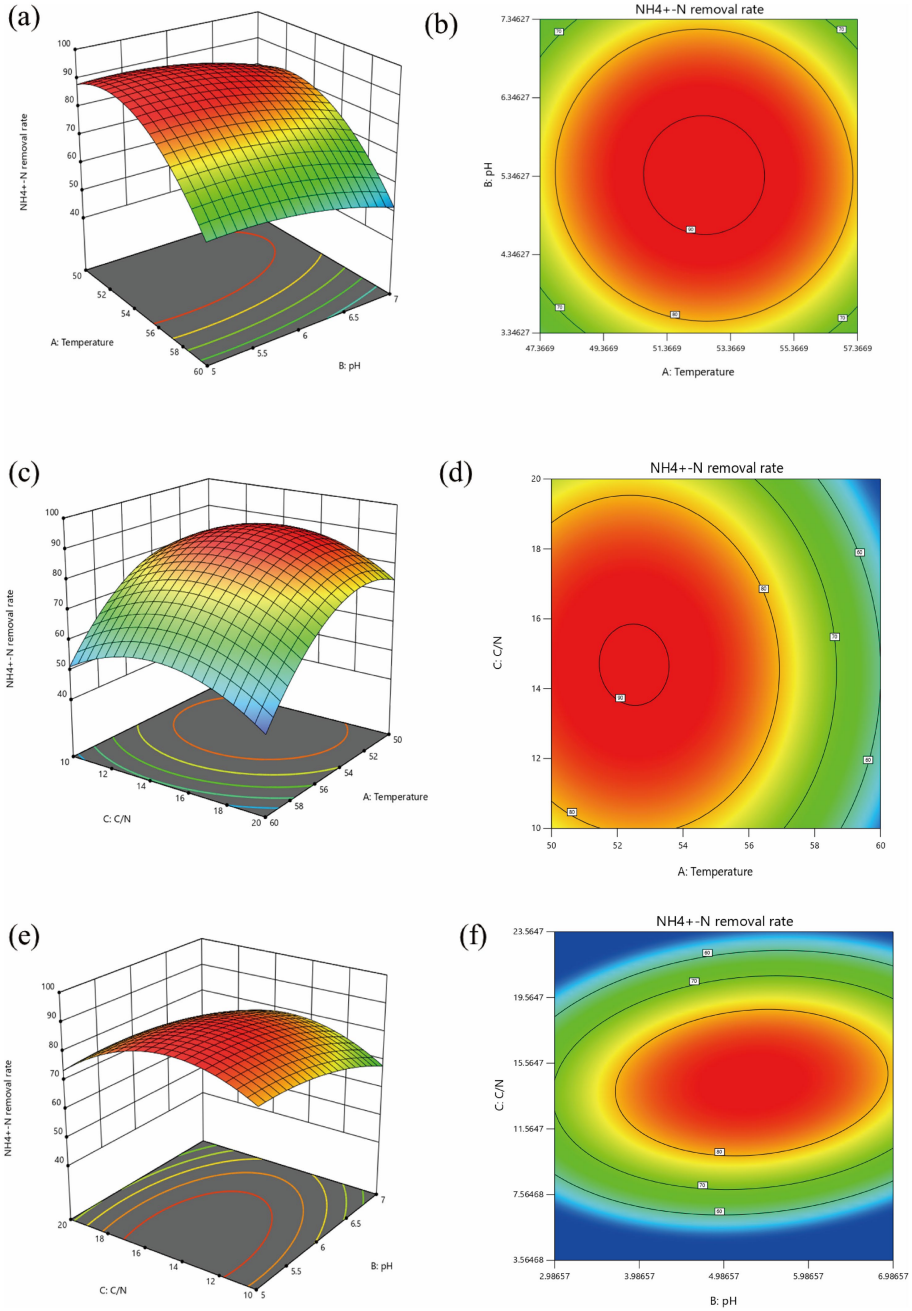
Different factors interacted with each other to affect the response surface diagram and corresponding contour lines of GW7 to NH_4_^+^-N removal efficiency. Temperature and pH **(a,b)**; Temperature and C/N ratio **(c,d)**; pH and C/N ratio **(e,f)**.

As illustrated in Figure 6, the response surface plots and corresponding contour lines depicted the effects of various factors on the NH₄^+^-N utilization efficiency by the high-temperature heterotrophic nitrifying consortium. From Figures 6a,b, change more steeply along the temperature axis compared to the pH axis, indicating that temperature had a greater impact on NH₄^+^-N utilization efficiency than pH. At a fixed pH, utilization efficiency initially increased and subsequently decreased with rising temperature In contrast, at a fixed temperature, variations in pH had only minor effects on NH₄^+^-N utilization efficiency.

As illustrated in Figures 6c,d, the contour lines changed more rapidly along the temperature axis compared to the C/N axis. This observation further indicated that temperature had a more pronounced effect on the response value than C/N. At a constant C/N, the utilization efficiency initially increased and then decreased as the temperature rose. Conversely, when temperature was held constant, changes in the C/N ratio had minimal effects on NH₄^+^-N utilization efficiency.

As shown in Figures 6e,f, the response surface was nearly flat, with contour lines showing similar changes along both the C/N and pH axes. This indicated that C/N and pH had a relatively minor impact on the response, and their interaction did not significantly affect NH₄^+^-N utilization efficiency. Overall, the relative importance of the three variables influencing NH₄^+^-N utilization efficiency was as follows: temperature > C/N > pH.

#### 3.2.3 Validation of response surface results

The analysis of the constructed regression model revealed that the optimal cultivation conditions for consortium were a C/N of 15:1, a temperature of 53°C, and an initial pH of 6. Under these conditions, the predicted NH₄^+^-N utilization efficiency was 87.80%. Three parallel experiments were conducted under these optimal conditions, and the relative error between the actual measured values and the model-predicted values was only 0.1%, indicating that the model accurately simulated the experimental results.

Through single-factor and response surface optimization experiments, the NH₄^+^-N utilization efficiency of consortium GW7 increased by 7.83%, and the OD_600_ ≥ 10^8^ CFU/mL. Experimental results indicated that the optimal cultivation conditions for consortium GW7 (pH 5–7, temperature 50–60°C) closely matched the requirements of practical composting processes (pH 5.5–8.5, temperature >50°C). Regarding the C/N, the optimal range under cultivation conditions was between 10 and 20, lower than the typical optimal range for practical composting (C/N 20–30). However, at a C/N of 20, the consortium maintained an NH₄^+^-N utilization efficiency of 80.36%, suggesting good adaptability to the lower limit of real composting conditions and indicating considerable potential for practical application. Furthermore, the optimal carbon source in laboratory conditions was sodium succinate, whereas practical composting commonly employs straw (mainly cellulose and hemicellulose). Furthermore, although the optimal carbon source in the laboratory was sodium glutarate, actual composting processes commonly used straw (cellulose and hemicellulose), and the composting environment was more complex. Therefore, the results obtained under laboratory conditions might have differed from those in the actual composting process. To ensure a smooth transition from laboratory research to practical application, we planned to validate the suitability of the consortium GW7 through pilot-scale and field composting trials in future studies.

### 3.3 Nitrogen transformation characteristics of high-temperature-tolerant heterotrophic nitrifying consortium GW7

**Figure 7:**
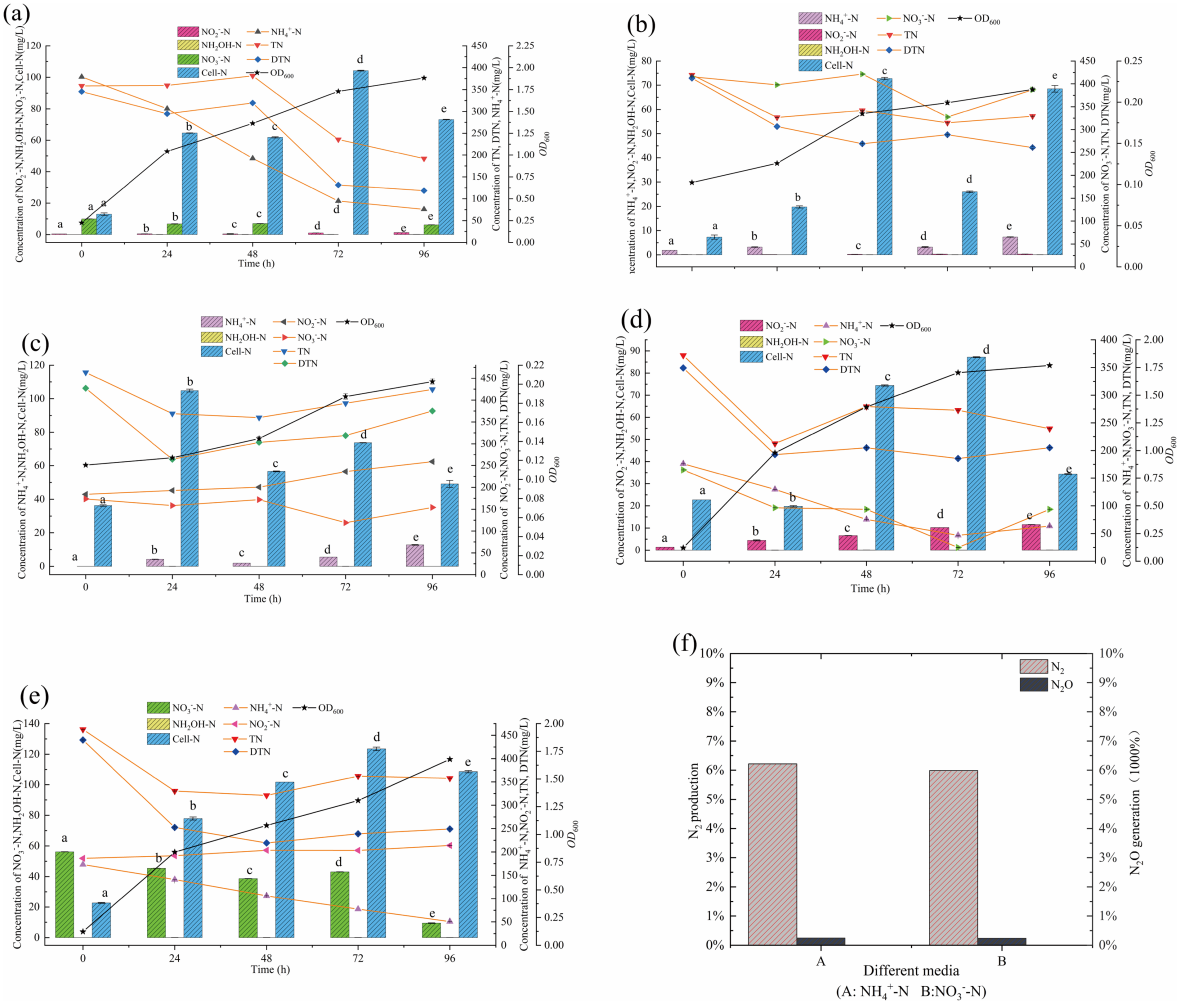
Growth and nitrogen transformation of consortium GW7 with NH_4_^+^-N **(a)**, NO_3_^−^-N **(b)**, NO_3_^−^-N and NO_2_^−^-N **(c)**, NH_4_^+^-N and NO_3_^−^-N **(d)**, NH_4_^+^-N and NO_2_^−^-N **(e)** as the only nitrogen source; **(f)** N_2_ and N_2_O production of thermophilic consortium GW7. Different lowercase letters in the figure indicate significant differences (*p* < 0.05).

When high-temperature heterotrophic nitrifying consortium GW7 was provided with NH_4_^+^-N as the sole nitrogen source (Figure 7a), the OD_600_ increased to 1.88 after 96 h during the logarithmic growth phase. As consortium GW7 grew rapidly, the NH_4_^+^-N concentration gradually decreased. By the end of the 96 h cultivation period, 303.01 mg/L of NH_4_^+^-N was utilized, with a utilization efficiency of 79.97%. Cell-N increased from an initial concentration of 12.92 mg/L to 73.13 mg/L after 96 h (*p* < 0.01). When NH_4_^+^-N and NO_3_^−^-N were provided as mixed nitrogen sources (Figure 7d), the NH_4_^+^-N utilization efficiency was 73.40%. From 0 to 72 h, NH_4_^+^-N concentration decreased, and Cell-N increased from 22.68 mg/L to 34.36 mg/L (*p* < 0.01). In the case of NH_4_^+^-N and NO_2_^−^-N as mixed nitrogen sources (Figure 7e), the NH_4_^+^-N utilization efficiency was 70.06%. From 24 to 72 h, NH_4_^+^-N concentration declined, and Cell-N reached a maximum of 123.48 mg/L. The above nitrogen concentration changes indicated that consortium GW7 preferentially assimilated NH_4_^+^-N when nitrogen was abundant, likely through the GS and GOGAT pathways, converting NH_4_^+^-N into essential nitrogen compounds like amino acids, proteins, and nucleic acids to support growth and reproduction (Pal et al., 2014; Fu et al., 2023).

When NH_4_^+^-N was the sole nitrogen source, there was no significant accumulation of NO_3_^−^-N or NO_2_^−^-N after 96 h, and Cell-N significantly increased after 72 h. This suggested that consortium GW7 performed nitrification, converting NH_4_^+^-N to NO_3_^−^-N. The NO_3_^−^-N is then likely reduced via the assimilatory nitrate reduction pathway, transforming it into NH_4_^+^-N, which was subsequently utilized by the strain. Although N_2_O and N_2_ were detected in the culture medium containing NH_4_^+^-N as the only nitrogen source, the production of N_2_O was 0.15%, and the N_2_ production was 5.98% (Figure 7f), which was significantly lower than the gas production of the nitrifying strain *Halomonas venusta* SND-01 (27.58%) (Huang et al., 2023). Heterotrophic nitrifying bacteria do not generate energy through heterotrophic nitrification, so they typically need to couple with aerobic denitrification to handle the excess reducing power generated during nitrification (Fang et al., 2024).

The heterotrophic nitrifying consortium GW7, when using NO₃^−^-N as the sole nitrogen source (Figure 7b), showed an OD_600_ growth and a nitrate utilization efficiency of 21.18% within 0–96 h. At 96 h, an increase in NH₄^+^-N was observed, with no accumulation of NO₂^−^-N during this period. Cell-N increased from 7.30 mg/L to 68.47 mg/L (*p* < 0.01). When using a mixed nitrogen source of NO₂^−^-N and NO₃^−^-N (Figure 7c), NO₃^−^-N decreased by 52.53 mg/L between 48–72 h, NO₂^−^-N increased by 35.69 mg/L, and NH₄^+^-N concentration rose by 3.66 mg/L, while Cell-N increased by 17.18 mg/L. When NH₄^+^-N and NO₃^−^-N were used as mixed nitrogen sources (Figure 7d), the nitrate utilization efficiency was 43.18%, with NO₃^−^-N rapidly decreasing from 164.93 mg/L to 24.90 mg/L within 72 h (*p* < 0.01), while NO₂^−^-N increased by 10.19 mg/L, and Cell-N reached a maximum of 87.19 mg/L. When NH₄^+^-N and NO₂^−^-N were used as nitrogen sources (Figure 7e), NO₂^−^-N accumulation efficiency reached 37.59% after high-temperature cultivation for 96 h, with continued increase in NO₂^−^-N concentration. These nitrogen changes suggested that the heterotrophic nitrifying consortium GW7 likely follows the ANRA pathway, where NO₃^−^-N was reduced by nitrate reductase as an electron acceptor, first converting NO₃^−^-N to NO₂^−^-N, followed by assimilation to form NH₄^+^-N, which was further utilized for cell growth, amino acid, and protein synthesis (Ren et al., 2023).

In summary, based on the changes in nitrogen concentrations and the production of gaseous products under different nitrogen sources, the nitrogen metabolism characteristics of the consortium GW7 can be inferred as follows: on one hand, the consortium assimilated NH₄^+^-N and converted it into organic nitrogen; on the other hand, it was also capable of nitrifying NH₄^+^-N to NO₃^−^-N, and subsequently following the assimilatory nitrate reduction pathway to convert NO₃^−^-N back to NH₄^+^-N, which was then further utilized for cell growth, as well as amino acid and protein synthesis.

### 3.4 Enzyme activity analysis

**Figure 8:**
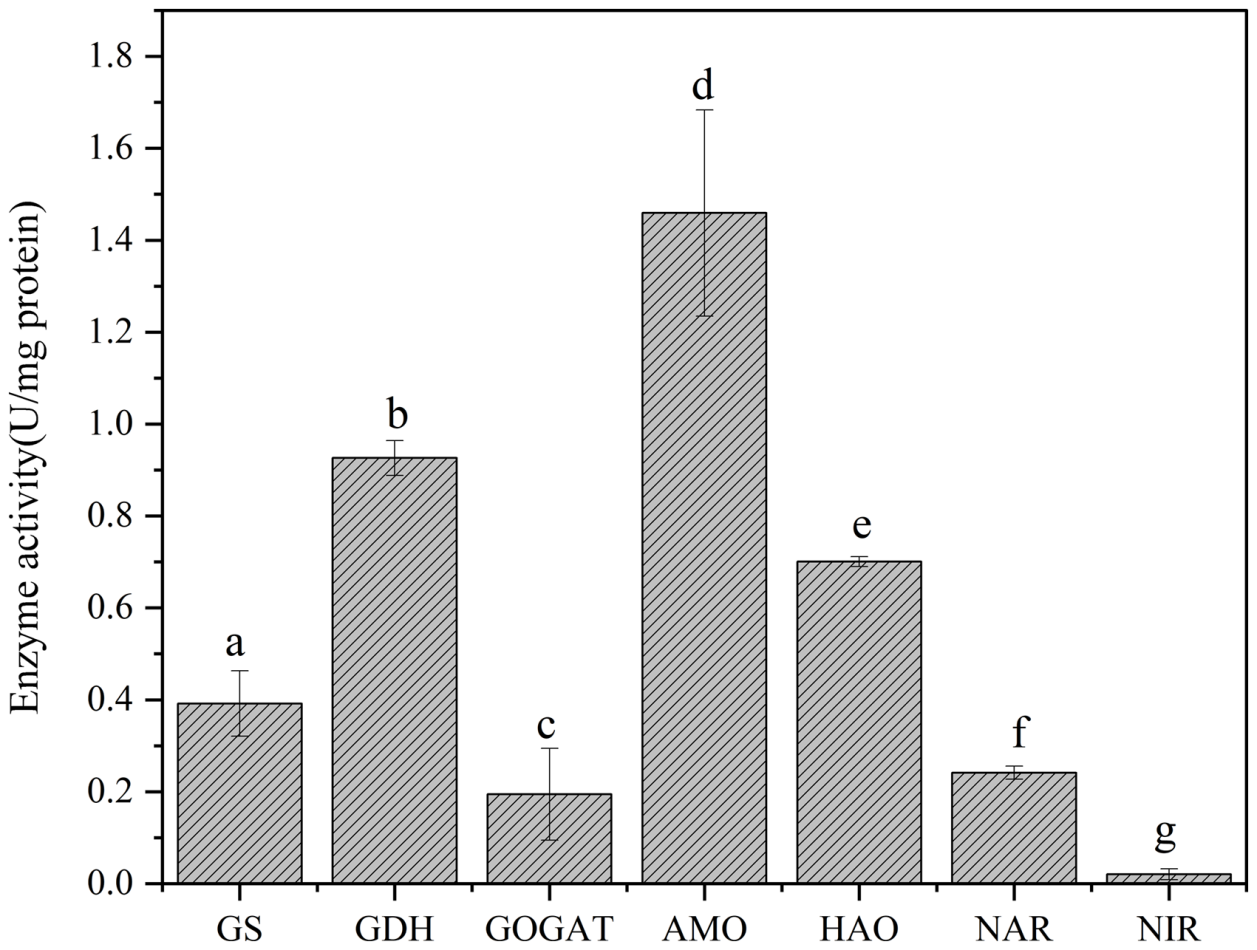
Key enzyme activities of thermophilic consortium GW7. Different lowercase letters in the figure indicate significant differences (*p* < 0.05).

In heterotrophic nitrifying bacteria, the key enzyme activities directly influences the nitrogen transformation process (Wu et al., 2024). To further elucidate the nitrogen pathways of heterotrophic nitrifying consortium GW7, this study measured the several key enzymes activities involved in nitrogen metabolism (Wang et al., 2022). As shown in Figure 8, the specific enzyme activities of AMO, HAO, nitriate reductase (NAR), and Nitrite reductase (NIR) in consortium GW7 were 1.459, 0.701, 0.0241, and 0.020 U/mg, respectively. The high activities of AMO and HAO indicated that consortium GW7 has a strong ability to oxidize ammonia, efficiently converting NH₄^+^ into NO₂^−^-N. while NAR (0.024 U/mg) and NIR (0.020 U/mg) exhibited much lower activity. Trace N₂ detection after 96 h confirmed limited denitrification capacity, indicating GW7 primarily functions as an efficient nitrifier with minimal concurrent denitrification.

GS converts NH₄^+^-N into glutamine, thereby fixing NH₄^+^-N into organic molecules; GDH catalyzes the incorporation of ammonia into glutamate, participating in nitrogen assimilation and metabolism; and GOGAT fixes ammonia onto glutamate to form glutamine (Muro-Pastor et al., 2005). The enzyme activities of GS, GDH, and GOGAT in consortium GW7 were detected as 0.392 U/mg, 0.926 U/mg, and 0.195 U/mg, respectively. The relatively high specific activity of GDH in consortium GW7 indicated that under conditions with sufficient initial nitrogen sources, GDH played a key role in regulating nitrogen balance by catalyzing the dehydrogenation of glutamate into α-ketoglutarate, thereby preventing excessive nitrogen accumulation. The lower activity of GOGAT compared to GS and GDH suggested that the GOGAT pathway was typically activated under nitrogen-limiting conditions, allowing the bacteria to utilize and store nitrogen (Sun et al., 2023). This finding supported the hypothesis that consortium GW7 primarily relied on the GDH pathway to directly convert ammonia and α-ketoglutarate into glutamate when sufficient ammonia was available in the environment (Zhu et al., 2023a, 2023b), further confirming the ammonium assimilation activity of heterotrophic nitrifying consortium GW7.

### 3.5 Nitrogen transformation pathways in high-temperature-tolerant heterotrophic nitrifying consortium GW7

**Figure 9:**
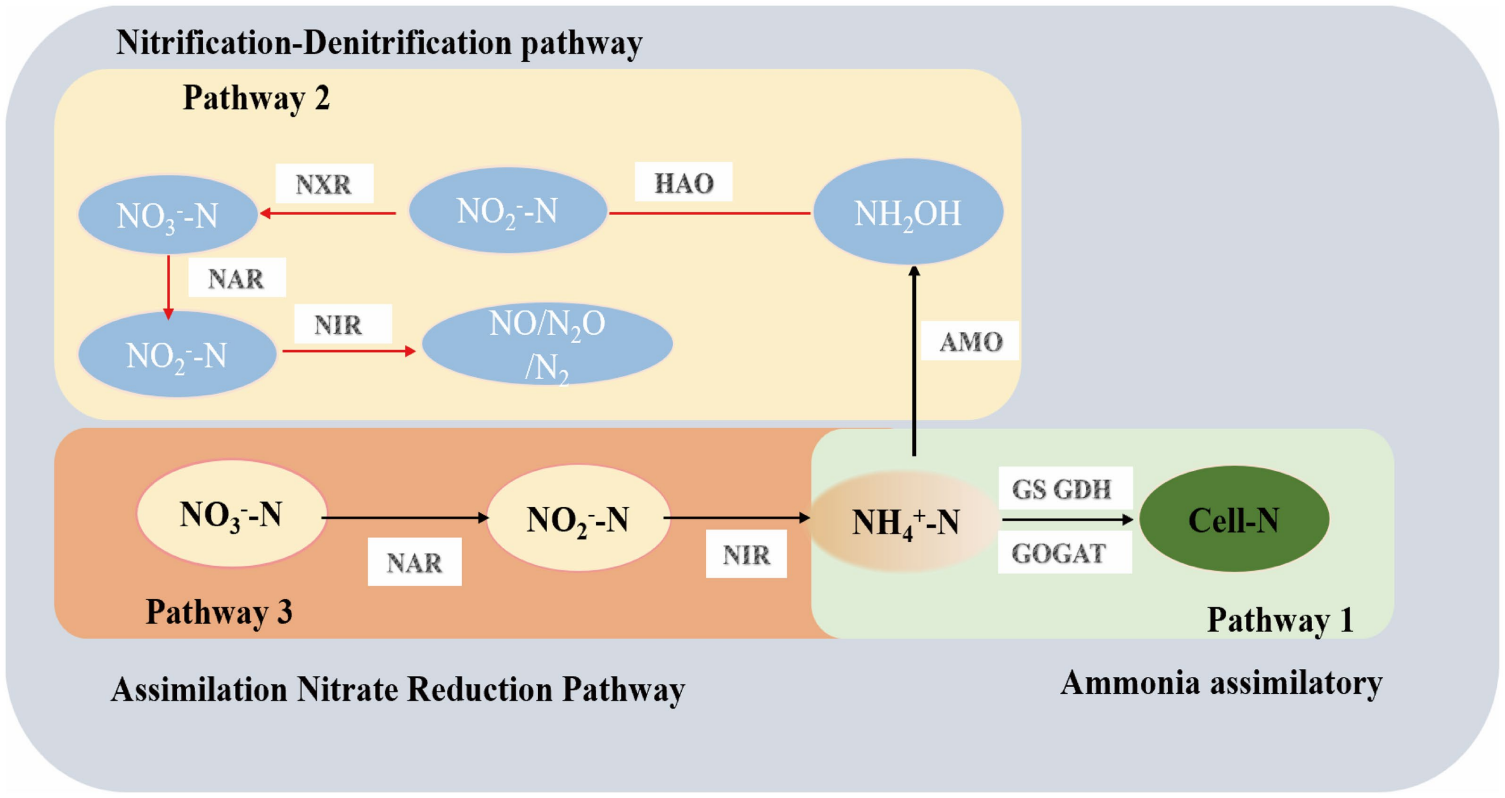
Possible nitrogen transformation pathway of thermophilic consortium GW7.

Based on the nitrogen transformation performance of consortium GW7 under different nitrogen sources, gas production, and the analysis of key enzyme activities in nitrogen pathways, the nitrogen metabolism pathways of consortium GW7 were identified as follows (Figure 9).

1. Assimilation (pathway 1): When NH₄^+^-N was abundant in the environment, consortium GW7 assimilates part of the NH₄^+^-N. The NH₄^+^-N was incorporated into cellular nitrogen sources such as amino acids and proteins, supporting cell growth and metabolism. During the composting process, NH₄^+^-N was one of the key nitrogen sources for composting microorganisms. Through assimilation, NH₄^+^-N was converted into cellular nitrogen sources (e.g., amino acids, proteins), which helps prevent the volatilization of ammonia gas, reducing its negative environmental impact (Maeda et al., 2011).
2. Heterotrophic nitrification (pathway 2): Some of the NH₄^+^-N was converted into NH₂OH and then further oxidized to NO₂^−^-N, which was subsequently transformed into NO₃^−^-N. Through heterotrophic nitrification, NH₄^+^-N in compost was gradually converted into NO₂^−^-N and NO₃^−^-N, effectively reducing ammonia volatilization and thereby minimizing nitrogen loss. This process improved nitrogen utilization efficiency (Chen et al., 2022).
3. Assimilatory nitrate reduction (pathway 3): In addition to assimilating NH₄^+^-N, consortium GW7 assimilated NO₃^−^-N and NO₂^−^-N through the assimilatory nitrate reduction pathway to convert them back into NH₄^+^-N. The NH₄^+^-N produced was then utilized for cell growth, amino acid, and protein synthesis. By assimilating NO₃^−^ and NO₂^−^, composting microorganisms were able to recover more nitrogen, reduce nitrogen loss, and support microbial growth by converting it into NH₄^+^-N, thereby regulating the nitrogen cycle in compost (Zhu et al., 2023b). In summary, high-temperature heterotrophic nitrifying consortium GW7 promoted efficient nitrogen transformation and demonstrates strong potential for practical applications.

## 4 Discussion

### 4.1 Study on the thermotolerance mechanism of consortium GW7

In this study, a thermophilic heterotrophic nitrifying consortium GW7, was successfully screened and demonstrated robust growth and high nitrification capability at 55°C. The mechanisms underlying its thermotolerance primarily involved a synergistic interplay among microbial composition, nitrogen transformation pathways, and nitrogen-metabolizing enzymes.

Firstly, the predominant genus in consortium GW7 was identified as *Aeribacillus* (82.6%), known for its ability to form spores. These spores remain metabolically dormant and are highly resistant to thermal stress (den Besten et al., 2017), thus protecting bacterial cells from structural damage under high-temperature conditions (Fan et al., 2019). Typically, bacteria within this genus tolerate temperatures between 55°C and 65°C (Tu et al., 2021), with certain strains even surviving above 100°C (González et al., 1999).

Secondly, consortium GW7 utilized multiple nitrogen transformation pathways that collectively sustain nitrogen metabolism and thermotolerance under elevated temperatures. The ammonia assimilation pathway ensures continuous nitrogen acquisition and utilization, thereby maintaining cellular physiological functions and metabolic activities at high temperatures (Chen et al., 2024). Nitrification pathway maintains nitrogen cycling balance, reduces ammonia accumulation, and provides additional energy for cellular metabolism (Zhang et al., 2023). Additionally, assimilatory nitrate reduction pathways help maintain intracellular redox balance and mitigate oxidative stress induced by high temperatures (Khanna et al., 2022).

In addition, the thermotolerance of key enzymes was also an important factor for the efficient nitrogen conversion of the GW7 microbial community under high-temperature conditions. AMO and HAO were key enzymes in the nitrification process, and their activity directly influenced the oxidation efficiency of NH₄^+^. The relatively high activity of AMO and HAO may have been due to temperature-induced allosteric effects, maintaining high catalytic efficiency and ensuring the smooth progression of the nitrification process (Tucci and Rosenzweig, 2025). Studies showed that AMO and HAO in certain thermophilic strains contained more hydrophobic amino acid residues, which helped form a more stable protein structure, thus preserving their activity at high temperatures (Liao et al., 2021). Furthermore, the thermal stability of these enzymes might also have been related to the protein folding and conformational stability. Proper folding and conformational stability could have prevented enzyme denaturation at high temperatures (Li et al., 2005). Relatively lower activities of NAR and NIR might have minimized oxidative damage by facilitating low-level denitrification processes to preserve intracellular redox homeostasis (Kessler et al., 2013). The high activity of GDH also helped the microbial community assimilate ammonia into organic nitrogen compounds, maintaining stable nitrogen metabolism under high temperatures, thereby enhancing the thermotolerance of the consortium GW7 (Meng et al., 2016).

### 4.2 Nitrogen metabolism pathway analysis of high-temperature-tolerant heterotrophic nitrifying consortium GW7

Based on the capacity of consortium GW7 to utilize diverse inorganic nitrogen sources and the measured activities of key nitrogen-transforming enzymes, we conclude that GW7 employed multiple metabolic pathways, including ammonia assimilation, assimilatory nitrate reduction pathway, and heterotrophic nitrification. Studies have shown that some heterotrophic nitrifying bacteria can simultaneously perform nitrification and denitrification processes while assimilating NH₄^+^ and NO₃^−^. These bacteria exhibit complex metabolic modes, incorporating assimilation, nitrification, and denitrification (Zhang et al., 2022).

In the ammonia assimilation pathway, the GDH enzyme of consortium GW7 showed significantly higher activity compared to GS and GOGAT. GS and GDH catalyze the formation of glutamine and glutamic acid, integrating NH₄^+^ into the organic nitrogen pool (Meng et al., 2016). Although GS has a higher affinity for ammonia (with a Km in the micromolar range), GDH exhibits a lower affinity (Km in the millimolar range) (Yoneyama and Suzuki, 2018). This difference makes GDH more effective in environments with high ammonia concentrations, while GS is crucial in ammonia-limited environments (Yoneyama and Suzuki, 2020). In this study, the initial NH₄^+^-N concentration was 400 mg/L, and the high initial nitrogen concentration led to significantly higher GDH enzyme activity, likely due to the nitrogen-rich conditions, where ammonia is directly assimilated through GDH rather than glutamine synthesis. In ammonia-rich environments, microorganisms enhance GDH synthesis or activity to convert ammonia into non-toxic glutamic acid, thus maintaining nitrogen balance and cellular health (Elsanosi et al., 2024). Ammonia fixation into organic nitrogen also avoids the production of greenhouse gases, in contrast to nitrification-denitrification processes.

The AMO enzyme activity of consortium GW7 was 1.459 U/mg, much higher than that of *Bacillus thuringiensis* WXN-23 (0.11 U/mg protein) (Xu N. et al., 2021). The high activity of AMO further confirms the strong heterotrophic nitrification ability of consortium GW7, enabling it to achieve an NH₄^+^-N removal efficiency of 87.80%. This also indicates that AMO plays a dominant role in ammonia oxidation, where NH₄^+^-N is first converted to NH₂OH-N under the catalysis of AMO. The study of HAO in crude enzyme extracts of consortium GW7 showed that its HAO enzyme activity (0.701 U/mg) was significantly higher than that of *Klebsiella pneumoniae* CF-S9 (0.051 U/mg) (Padhi et al., 2013). This suggests that under sufficient NH₄^+^-N, consortium GW7 not only conducts ammonia assimilation but also exhibits excellent nitrification capabilities, converting NH₄^+^ into NO₃^−^, making nitrogen more available for plants or microorganisms (Huang et al., 2020). The bacterial consortium GW7 can also convert the nitrate produced by nitrification back to NH₄^+^-N through the assimilatory nitrate reduction pathway. Similar to the strain *Halomonas venusta* SND-01, which also established a complex pathway including ammonia assimilation, heterotrophic nitrification, and assimilatory nitrate reduction, in the assimilatory nitrate reduction pathway, nitrate is reduced to nitrite by the assimilatory nitrate reductase encoded by the *nasAB* genes and further reduced to ammonia by the assimilatory nitrite reductase encoded by the *nirA* gene (Huang et al., 2023).

In nitrogen- and energy-rich environments, heterotrophic nitrifying bacteria can flexibly select suitable metabolic pathways, adjusting the balance between nitrification and nitrogen assimilation in response to external conditions. This adaptability enhances their survival and nitrogen transformation efficiency (Huang et al., 2023). The efficient use of NH₄^+^-N by consortium GW7 demonstrates its potential to improve nitrogen transformation during aerobic composting by promoting nitrification, thereby reducing the loss of harmful nitrogen compounds such as ammonia gas (Kuypers et al., 2018). Moreover, its ammonia and nitrate assimilation capabilities allow for better regulation of nitrogen transformation and source utilization during composting, minimizing the loss and accumulation of NH₄^+^-N and nitrate, and improving nitrogen balance.

### 4.3 Advantages and application potential of consortium GW7 in aerobic composting

Heterotrophic nitrifying bacteria exhibit various advantages, including a wide range of isolation sources, rapid growth rates, and strong environmental adaptability, enabling them to effectively reduce nitrogen losses, inhibit odor emissions, and enhance compost quality (Zhao et al., 2010). In this study, the heterotrophic nitrifying consortium GW7 demonstrated remarkable ammonia nitrogen utilization capability in a thermophilic composting system. At 55°C with an initial NH₄^+^-N concentration of 400 mg/L, the NH₄^+^-N utilization efficiency reached 87.80%. This performance significantly surpasses previously reported thermophilic strains, such as *Brevibacillus agri* N2 with an NH₄^+^-N utilization efficiency of 45.47%, *Gordonia paraffinivorans* N52 with 51.8%, and *Anoxybacillus contaminans* HA with 71.0% (Zhao et al., 2022a, 2022b; Chen et al., 2015). It is worth noting that previous studies reported a lower ammonia nitrogen removal capacity in thermophilic bacterial strains, which could be attributed to their limited metabolic pathways and the insufficient activity of key enzymes under high-temperature conditions (Zhao et al., 2022a). For instance, the strain *Brevibacillus agri* N2 relied solely on a single nitrification pathway, and its key enzymes, such as AMO, exhibited reduced activity under high-temperature conditions, thus limiting nitrogen transformation efficiency. In contrast, the consortium GW7 not only possessed multiple nitrogen transformation pathways but also demonstrated high activity of key enzymes.

During the thermophilic phase of composting, mesophilic nitrifying bacteria often fail to adapt to elevated temperatures, resulting in significantly inhibited growth performance and reduced nitrogen transformation efficiency. For example, strain *Acinetobacter* sp. Y1 exhibited a notable decline in NH₄^+^-N utilization efficiency at 45°C (Liu et al., 2019), while strain *Pseudomonas* sp. JQ170 showed NH₄^+^-N utilization efficiency at 50°C that was less than one-third of its efficiency at optimal growth temperatures (Xu S. et al., 2021). In contrast, the exceptional thermotolerance of consortium GW7 enabled it to thrive under high-temperature composting conditions (typically ranging from 50 to 70°C), effectively mitigating ammonia volatilization and overcoming the reduced activity typically observed in mesophilic strains during the thermophilic stage. This ensured consistent functional stability in practical applications. Furthermore, as a stable microbial consortium, GW7 exhibited superior environmental adaptability and functional robustness compared to single-strain inoculants (Wang et al., 2024b; Liu et al., 2023), highlighting its considerable potential for practical composting applications.

During aerobic composting, easily degradable organic nitrogen compounds (e.g., proteins and amino acids) are rapidly mineralized into NH₄^+^ via microbial activity in the initial composting stage. Subsequently, significant nitrogen losses primarily occur due to NH₄^+^ volatilization during the thermophilic phase (Xiao et al., 2017). In this critical high-temperature phase, consortium GW7 exhibited notable advantages by possessing multiple nitrogen transformation pathways (ammonia assimilation, heterotrophic nitrification, and assimilatory nitrate reduction). These integrated metabolic pathways facilitated a synergistic interaction between assimilation and nitrification processes, substantially enhancing nitrogen conversion efficiency. This effectively overcame the limitations associated with single-strain metabolism, significantly reduced NH₄^+^ volatilization losses, and addressed the key challenge of substantial nitrogen losses during the high-temperature stage of traditional composting.

## 5 Conclusion

The thermophilic heterotrophic nitrifying consortium GW7, enriched from compost samples, shows strong potential for nitrogen transformation at 55°C. Consortium GW7 can simultaneously utilize NH₄^+^-N and NO₃^−^-N, assimilating NH₄^+^-N via ammonia assimilation and converting excess NH₄^+^-N into NO₃^−^-N through heterotrophic nitrification. It can also reduce NO₃^−^-N back to NH₄^+^-N via assimilatory nitrate reduction, integrating nitrogen cycle pathways. The NH₄^+^-N produced is assimilated into microbial biomass, supporting growth. These pathways highlight GW7’s adaptability and functional versatility in high-temperature environments. The consortium’s ability to enhance nitrogen retention and reduce ammonia emissions during aerobic composting makes it promising for improving nitrogen conservation in composting technologies. Subsequently, pilot trials will be conducted in industrial-scale composting facilities to evaluate the nitrogen retention effect of GW7 under practical conditions. The microbial regulatory mechanisms of GW7 will be investigated, and composite microbial inoculants will be developed, leveraging the complementary functions of different microbial strains to enhance overall compost quality and reduce nitrogen loss.

## Data Availability

The original contributions presented in the study are included in the article/Supplementary material, further inquiries can be directed to the corresponding authors.
