# Supplementary material for: Research on nitrogen transformation pathways of a thermophilic heterotrophic nitrifying bacterial consortium GW7
Source: Front Microbiol. 2025 Jun 30;16:1578865. doi: 10.3389/fmicb.2025.1578865 (PMC12257491; doi:10.3389/fmicb.2025.1578865)
Supplement: Supplementary file 1 [file Supplementary_file_1.docx]

**2 Materials and Methods**

**2.1 The enrichment of** **bacterial** **consortium**

(1) Sample Collection: Compost samples comprising cattle and sheep manure were collected from Guanghe County, Linxia Hui Autonomous Prefecture, Gansu Province, China. Samples were selected from the high-temperature phase (with temperatures exceeding 55°C) to ensure the presence of active thermophilic microorganisms. A five-point composite sampling method was applied to ensure representativeness. Samples were immediately transferred into sterile sampling bags, transported to the laboratory in insulated foam boxes containing ice packs, and stored at 4°C. Enrichment cultures were initiated within 24 h of sampling.

(2) Consortium Enrichment: For primary enrichment, approximately 2 g of compost sample was inoculated into 250 mL Erlenmeyer flasks containing 200 mL sterilized heterotrophic nitrification medium (HNM). Cultures were incubated at 55°C with continuous shaking (180 rpm) for 3–4 days. Subsequently, 10% (v/v) aliquots of enriched cultures were transferred to fresh HNM and cultured under identical conditions. This subculturing process was repeated 4–5 times to achieve stable microbial consortia.

(3) Subculturing and Maintenance: After achieving stable enrichment (typically five cycles), subculturing was routinely performed by aseptically transferring 1% (v/v) of the culture into fresh HNM once the optical density at 600 nm (OD₆₀₀) reached 1.0. This serial passaging was continuously conducted at 55°C and 180 rpm to maintain consortium viability and nitrification capacity.

(4) Primary Screening: After 96 h incubation in HNM, preliminary assessment of heterotrophic nitrification activity was conducted through qualitative assays: (i) Nitrite production was detected by adding Griess reagent, where purple/red coloration indicated NO₂⁻-N presence. (ii) Ammonium utilization capacity was qualitatively evaluated using Nessler’s reagent following tartaric acid pretreatment, where the intensity of color correlated with NH₄⁺-N consumption.

(5) Secondary Screening: Candidate consortia identified from primary screening were inoculated into screening medium with defined initial NH₄⁺-N concentrations. Following 96 h incubation at 55°C and 180 rpm, cultures were centrifuged at 5,500 rpm for 5 min at 4°C. Residual NH₄⁺-N concentrations in supernatants were quantified, and consortia exhibiting superior ammonia utilization were selected and preserved on agar slants at 4°C.

(6) High-Throughput Sequencing: Taxonomic composition of the optimal consortium GW7 was analyzed via 16S rRNA gene amplicon sequencing using the Illumina sequencing platform. Consortium GW7 demonstrated stable and efficient heterotrophic nitrification capability, primarily dominated by the genus Aeribacillus (82.6%). The GW7 consortium is maintained at the College of Animal Science and Technology, Gansu Agricultural University, under cryopreservation conditions at -80°C.
